# 40 ‘wild’ years: the current reality and future potential of assisted reproductive technologies in wildlife species

**DOI:** 10.1590/1984-3143-AR2024-0049

**Published:** 2024-08-26

**Authors:** Gabriela Mastromonaco

**Affiliations:** 1 Reproductive Sciences Unit, Toronto Zoo, Toronto, Ontario, Canada

**Keywords:** assisted reproduction, embryo technologies, biodiversity conservation, wildlife species

## Abstract

Over the past 40 years, assisted reproductive technologies (ARTs) have grown significantly in scale and innovation, from the bovine embryo industry’s shift from in vivo derived to in vitro produced embryos and the development of somatic cell-based approaches for embryo production. Domestic animal models have been instrumental in the development of ARTs for wildlife species in support of the One Plan Approach to species conservation that integrates in situ and ex situ population management strategies. While ARTs are not the sole solution to the biodiversity crisis, they can offer opportunities to maintain, and even improve, the genetic composition of the captive and wild gene pools over time. This review focuses on the application of sperm and embryo technologies (artificial insemination and multiple ovulation/in vitro produced embryo transfer, respectively) in wildlife species, highlighting impactful cases in which significant progress or innovation has transpired. One of the key messages following decades of efforts in this field is the importance of collaboration between researchers and practitioners from zoological, academic, governmental, and private sectors.

## Introduction

**Figure 1:**
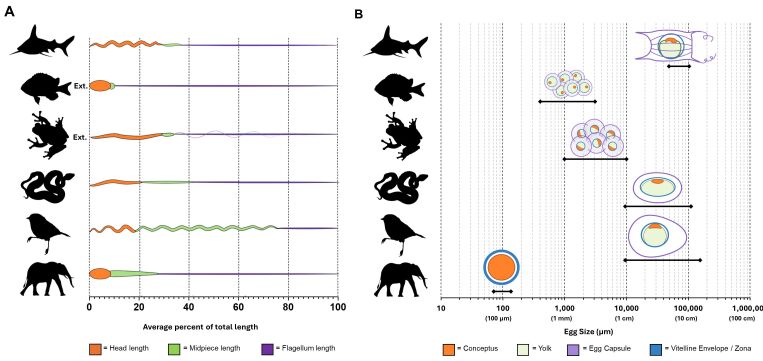
Diversity in gamete morphology across vertebrate taxa. Stylized sperm and egg drawings depict broad similarities and differences among Chondrichthyes, Osteichthyes, Amphibia, Reptilia, Aves, and Mammalia. There are further species-specific differences that are not captured in this schematic. (A) proportion of sperm head, midpiece and tail lengths among vertebrate classes. Total sperm lengths range from 20 – 350 µm depending on the species. Ext. = external; only sperm morphology from external (and not internal) fertilizers are shown. Sperm figure modified from Kahrl et al. (2022) by removing sperm images from internal fertilizers for Osteichthyes and Amphibia; licensed under a Creative Commons Attribution 4.0 International License (CC BY 4.0; https://creativecommons.org/.licenses/by/4.0/); (B) cellular and extracellular layers encompassing the eggs (or oocytes) of different vertebrate classes (Menkhorst and Selwood, 2008). Egg diameters range from 100 – 100,000 µm depending on the species. Egg images were created with BioRender.com (agreement number TG26OWIEYQ). Silhouette illustrations were contributed by various authors under public domain license (CC0 1.0 license) from PhyloPic (http://phylopic.org).

Decreasing wildlife population trends are coupled with concerns of ecosystem disruption that have far-reaching impact on animals and humans alike. The International Union for Conservation of Nature Species Survival Commission’s (IUCN SSC’s) One Plan Approach to conservation promotes in situ and ex situ connectivity in population management to enhance species sustainability goals (Byers et al., 2013). Assisted reproductive technologies (ARTs) can have a significant impact on achieving species recovery targets through long-term storage of genetic material as assurance against on-going or future depletion of diversity. While ARTs are not the miracle solution for fighting extinction, they are a powerful supporting tool for genetic management across time. Interest in the application of ARTs in wildlife species began in the mid-1970s with the transfers of flushed ‘exotic’ embryos into recipients of related livestock species (Kydd et al., 1985; Dresser, 1986). They gained momentum in the mid-1980s with the landmark paper by Jonathan Ballou, a researcher focused on the genetic and demographic challenges of small population management (Ballou, 1984). The wide range of reproductive strategies across vertebrate taxa (see Figure 1 for sperm and oocyte diversity) significantly influenced the development of ART programs, and the importance of domestic animal models was recognized early on (Wildt et al., 1986). This review will touch briefly on the progress of artificial insemination (AI) as a conservation breeding tool and focus on the potential for embryo technologies, specifically multiple ovulation embryo transfer (MOET) and in vitro produced embryo transfer (IVPET), to meet the growing threats to species sustainability.

## Sperm Technologies: Impact of Artificial Insemination

The past century demonstrated the potential of sperm-focused technologies. In wildlife species, insemination of females under natural or stimulated cycles continues to be the most effective ART for offspring production. To date, artificial insemination (AI) has been attempted successfully in ~100 species across diverse taxa (Swanson and Penfold, 2018) with reptiles just beginning to benefit from an increased investment in the development of ARTs (Perry, 2021). Protocols for external fertilizers, such as fishes and amphibians, have also advanced, and will be discussed in the section below on in vitro embryo production. The power of AI stems from its widespread application due to the lower investment of resources (funds, specialized equipment, technical expertise), but also because the embryo is developing in its natural environment, precluding the need for in-depth knowledge of pre-implantation embryo development.

Sperm from wild mammals is collected by electroejaculation or post-mortem epididymal dissection, and more recently by urethral catheterization (Lueders et al., 2012; Prieto et al., 2014). The latter approach will be beneficial as animal welfare guidelines continue to change, making minimally invasive alternatives a necessity. In non-mammalian species, sperm samples are obtained by massage, artificial vagina, or catheterization (reviewed by Prieto et al., 2014). For many mammals, birds and fishes, sperm handling solutions are commercially available from related domestic animals and humans. In species where a commercial sperm extender is unavailable or not providing the desired outcomes, modification of a commercial product (e.g., by altering glycerol concentration) or optimization of a ‘home-made’ recipe is required.

Fresh/chilled sperm has produced viable offspring across all taxa (reviewed by Blanco et al. (2009) (birds); Kouba et al. (2009) (amphibians); Beirão et al. (2019) (fishes); Mastromonaco and Songsasen (2020) (mammals/birds); Perry (2021) (reptiles)); however, implementation of ARTs for long-term genetic management requires access to sperm over extended periods of time for the infusion of novel or minimally represented genes. As a result, sperm cryobiology has been an active field of study resulting in hundreds of published papers detailing the effects on pre- and post-thaw sperm characteristics. Sperm cryopreservation and use in ARTs have been successful in many of the species attempted, even if just a one-time success. Species exhibiting chilling sensitivity traits can be challenging despite extensive efforts to optimize species-specific techniques. In these cases, enhancement of standard protocols may provide additional protection to sperm membranes to improve post-thaw motility and integrity, such as the use of specialized density gradients or oviductal extracellular vesicles in cheetahs (*Acinonyx jubatus*) (Crosier et al., 2009; Ferraz et al., 2020). More recently, several researchers shifted their focus to freeze-drying sperm, and preliminary trials showed promising outcomes (Kaneko et al., 2014; Anzalone et al., 2018). The possibility of eliminating the dependence on liquid nitrogen makes this an attractive method for field collections and resource-restricted conservation biobanks.

Insemination of naturally cycling or hormonally primed females using either fresh, chilled or frozen-thawed sperm has been accomplished through a variety of methods: non-surgically using transcervical (mammals) / transcloacal (birds, reptiles) sperm deposition, or surgically using laparotomic or laparoscopic sperm deposition into the uterine cavity or oviduct (Swanson and Penfold, 2018). Notably, for AI to be successful, the female must be at the right stage of the ovarian cycle to receive the sperm, except perhaps in species with long-term sperm storage capabilities within the female (e.g., bats: 6 months; lizards: up to 1.5 years; sharks: 1-2 years; snakes: up to 7 years; Holt and Fazeli (2016). The important topic of ovarian stimulation will be discussed further in the embryo technologies section below.

One of the greatest challenges for wildlife ARTs is advancing the technique from research to application. With limited access to research animals and materials, protocol development and implementation can take multiple decades. Thus, many of the AI births to date have been proof-of-concept attempts that generated media attention without further progress. The slow advancement and relatively low success rates present a significant challenge when advocating for the inclusion of ARTs in conservation breeding programs. Despite these drawbacks, AI has been used not only to achieve ex situ targets (sustainability of captive populations), but also to support in situ needs (recovery of the species in their native ranges).

Spotlight: Common Bottlenose Dolphin (*Tursiops truncatus*)

Throughout the 2000s, Todd Robeck and Justine O’Brien spear-headed a comprehensive sperm-based approach that included fresh-chilled, frozen-thawed and sex-sorted sperm inseminations in bottlenose dolphins. AI offered an opportunity to overcome the challenges of managing the genetics and demographics of a closed captive population in a species that is difficult to translocate between institutions. Ovarian synchronization, manual semen collection and sperm cryopreservation protocols were developed, and intra-uterine inseminations resulted in 60-70% pregnancy rates for fresh-chilled, frozen-thawed un-sorted and frozen-thawed sex-sorted sperm (O’Brien and Robeck, 2006; Robeck et al., 2013). To date, 30 bottlenose dolphin calves have been born from AI using sex-sorted frozen-thawed sperm with a sex predetermination rate of 93% (United Parks & Resorts, 2024), making it one of the greatest achievements in wildlife AI. Building on this work, successful inseminations have resulted in pacific white-sided dolphin (*Lagenorhynchus obliquidens*; Robeck et al., 2009), orca (*Orcinus orca*; Robeck et al., 2004), and beluga whale calves (*Delphinapterus leucas*; O’Brien et al., 2008). Although ART development has primarily benefitted marine mammals in managed care, the groundwork has been laid for possible application in future wild cetacean recovery efforts.

## Embryo Technologies: Enhanced Potential for Genetic Rescue

With the on-going pressure to preserve and restore biodiversity, there is an urgency to include female genetics in the wildlife biobanks. This requires investment in embryo technologies, specifically the acquisition of a) oocytes for fertilization or cryopreservation, b) embryos for transfer or cryopreservation, and c) somatic cells for production of gametes and embryos. These techniques are dependent on species-specific knowledge of the female’s reproductive biology and the embryo’s early development. Despite the additional challenges, the need to ensure female genetic contribution to conservation populations has stimulated concentrated efforts in various threatened species.

The 1970s and 80s sparked an interest in interspecific transfers to investigate the possibility of using domestic animal surrogates to gestate endangered species embryos. These early attempts included the transfers of in vivo derived (IVD) embryos from Grant’s zebra (*Equus quagga boehmi*) and Przewalski’s horse (*Equua ferus przewalskii*) into domestic mare, bongo antelope (*Tragelaphus eurycerus*) into common eland antelope (*Taurotragus oryx*), and more (Dresser, 1988; Allen and Wilsher, 2020). The subsequent decades brought a re-focusing of attention onto basic reproductive biology to expand the knowledge of reproductive anatomy, physiology, and gamete biology (Pukazhenthi and Wildt, 2004; Herrick, 2019). Thus, while live births from embryo technologies have been documented, the outcomes have not been as consistent and widespread as AI, apart from fishes and amphibians. Currently, embryo technologies are not being implemented as a population management strategy in mammals, birds, or reptiles.

- Ovarian Cycle Monitoring and Control

The female component of ARTs requires monitoring or control of ovarian dynamics in the oocyte donors and embryo recipients. For offspring-endpoint AIs, both natural and ‘artificially’ synchronized (i.e., application of exogenous hormones or alteration of environmental conditions such as changes in temperature or humidity) cycles are used. When using natural cycles, as in the giant panda, non-invasive hormone monitoring (urine and feces) is a necessity since blood sampling and ultrasonography are not possible in species that cannot be routinely handled (Kersey et al., 2010). In contrast, many elephants are trained for blood collection allowing serum LH levels to be used to time inseminations during natural cycles, a feat that is possible only due to the double LH peaks in African and Asian elephants (*Loxodonta africana* and *Elaphas maximus*, respectively; Thitaram and Brown, 2018). Aside from the species that can be trained for blood sampling, the role of non-invasive hormone monitoring in conservation breeding programs cannot be underestimated. Fecal hormone profiles have helped shed some light on the complexities of embryonic diapause, follicular stasis, and more. Similarly, the use of transrectal ultrasound in animals that can be chute trained has been instrumental in elucidating ovarian dynamics and establishing the timing for inseminations and embryo transfers (Pennington et al., 2019).

Ovarian synchronization with exogenous hormones has been successfully applied in many mammalian species using protocols generally adopted from farm and laboratory animals with certain species-specific modifications to optimize outcomes. In wild felids, gonadotropin dosages based on domestic cat studies proved difficult to extrapolate to diverse wild cat species on size alone due to species-specific differences in sensitivities to eCG and hCG: ocelots (*Leopardus pardalis*; 9 kg) required double the dose than cheetahs (35 kg), whereas clouded leopards (*Neofelis* nebulosa; 15 kg) required the same dose as domestic cats (2 kg) (Thongphakdee et al., 2018). Studies in domestic cats and cheetahs demonstrated that progestin priming prior to gonadotropin administration normalized the endocrine response thereby improving ovulation, corpus luteum function and oocyte developmental potential (Stewart et al., 2012; Crosier et al., 2017).

Acquisition of multiple oocytes from donor females requires ovarian super-stimulation in monovulatory species, as well as certain polyovulatory species. Application of exogenous hormones (e.g., FSH) to enhance follicular recruitment must maintain a balance between producing a maximum number of follicles and ensuring the retrieval of competent oocytes. Domestic cattle hormone regimens have formed the basis of many wildlife super-stimulation trials, ranging from wild bovids (e.g., banteng (*Bos javanicus*)) to antelopes (e.g., addax (*Addax nasomaculatus*)) (Sontakke, 2018). Notably, studies in domestic cattle demonstrated that extrapolation between even the more closely related species and subspecies, namely holstein (*Bos taurus*), gir (*Bos indicus*), and murrah cattle (*Bubalus bubalis*), is problematic due to the inherent differences in follicular waves, antral follicle populations, and follicle and corpus luteum diameters (Baldrighi et al., 2022).

For non-mammalian species, similar approaches employing exogenous hormones have been documented. In amphibians, both spermiation and ovulation can be induced with injections of hCG or GnRH; however, in certain species such as the Puerto Rican crested toad, hormones alone were not effective in stimulating ovulation, and altered environmental conditions (e.g., hibernation at low temperatures) were required prior to hormone administration to enhance outcomes (Kouba and Vance, 2009). In both fishes and amphibians, addition of dopamine antagonists to GnRH protocols improved ovulation and fertilization (Van Eenennaam et al., 2008; Silla et al., 2021). In birds, on the other hand, inseminations have been timed to natural laying cycles (Swengel and Tuite, 1997), with alterations in photoperiod being used to induce egg laying outside of the breeding season (Zhu et al., 2017).

- Multiple Ovulation Embryo Transfer (MOET)

MOET has not been widely attempted in wild mammals. Retrieval of multiple embryos from naturally bred or artificially inseminated donor females are either transferred fresh to recipient females or cryopreserved for future use; an effective method for increasing a female’s lifetime reproductive output. Despite the abundance of data from domestic cattle studies highlighting the increased competence of IVD embryos compared to in vitro produced (IVP) embryos (Ferré et al., 2020), wildlife researchers interested in obtaining female genetics have focused on IVPET. Thus, aside from the early attempts on interspecies ET and efforts in species of commercial importance (e.g., dromedary camels (*Camelus dromedarius*), red deer (*Cervus elaphus*)), MOET has been applied in only a handful of species of socio-cultural, economic, and nutritional relevance to indigenous communities (wood bison (*Bison bison athabascae*), yak (*Bos grunniens*), llama (*Lama glama*), alpaca (*Vicugna pacos*); Mastromonaco, 2024). The lack of traction with MOET in conservation breeding programs has been due to difficulties in accessing and handling invaluable donor females. However, even in domestic cattle, there has been a shift away from MOET towards IVPET, with the number of transferable IVP embryos now surpassing the number of IVD embryos produced annually; a change that corresponds with the increasing efficiency of IVP systems (Ferré et al., 2020).

Spotlight: Wood Bison (*Bison bison athabascae*)

Since 2006, Gregg Adams has led a systematic plan to establish a biobank of disease-free sperm and embryos from free-ranging wood bison to mitigate the effects of tuberculosis and brucellosis in wild herds (Bison Integrated Genomics (BIG) Project, 2022). Domestic cattle synchronization and super-stimulation protocols have been somewhat effective in wood bison, particularly with modifications (i.e., single slow-release FSH dose) to reduce handling frequency and stress (Toosi et al., 2013). Interestingly, exogenous hormones could overcome seasonal constraints to produce competent oocytes with successful development in vitro but not in vivo, suggesting that differences in the oviductal environment negatively affected IVD embryo development in the anovulatory season (Palomino et al., 2020). Subsequent attempts to produce embryos across ovulatory and anovulatory seasons focused on retrieving oocytes for IVP (Zwiefelhofer et al., 2022a). To date, >20 calves have been born from AI, MOET and IVPET using fresh-chilled, frozen-thawed and sex-sorted sperm (reviewed by Acevedo and Barfield, 2023). With progress now being made on the collection of sperm and oocytes from free-ranging wood bison (Zwiefelhofer et al., 2022b), the wood bison project is one of the applications of the One Plan Approach to conservation that will include biobanking and ARTs; a potential model for the wisent, another bison species at risk (Duszewska et al., 2022).

c) In Vitro Produced Embryo Transfer (IVPET)

IVPET is the goal of many wildlife ART programs interested in preserving female genetics. Both sperm and oocytes are taken outside their ‘natural habitats’ and moved ex vivo into the culture dish requiring in-depth knowledge of gamete maturation, fertilization, and embryo development to ensure post-implantation success. Studies have highlighted the differences between IVP and IVD embryos, with the latter typically resulting in greater success post-thaw, -implantation and -birth (Ferré et al., 2020). While domestic and laboratory animal models have been instrumental in generating basic protocols, species-specific requirements have made advancement challenging in many wildlife species. Aquatic external fertilizers (fishes and amphibians), which release large numbers of oocytes and do not require the final embryo transfer step to produce live young, have experienced the greatest successes with IVP embryos. Aside from these cases, embryo technologies are not currently being implemented in species restoration programs. Wildlife IVP embryos have been created by both conventional (IVF) and advanced (somatic cell nuclear transfer; SCNT) methods to begin populating the conservation biobanks with female genetics (Mastromonaco, 2024). Optimistically, in vitro gametogenesis (IVG) may be contributing to the growing list of banked gametes and embryos in the coming years. To date, animals born from IVPET have been scarce such that announcements of confirmed pregnancies or births continue to generate media attention.

- In Vitro Fertilization (IVF)

Challenges with in vitro maturation, fertilization, and culture (IVM-IVF-IVC), typically reported as low polar body extrusion, cleavage and blastocyst rates in domestic species, are also experienced in many wildlife species (Mastromonaco, 2024). For instance, ovulation of immature (MI) oocytes and difficulties with in vitro maturation (<20% MII) in domestic dogs results in the need for in vivo matured oocytes in gray wolf (*Canis lupus*) IVPET trials (Nagashima and Songsasen, 2021). Similarly, a lack of optimal IVM systems for domestic cats (~60% MII) drives the outcomes in wild felid studies (Thongphakdee et al., 2020), and poor in vitro capacitation in domestic horse sperm leads to the use of intracytoplasmic sperm injection (ICSI) in zebra, Przewalski’s horse (Gambini et al., 2020), and white rhinoceros (Hildebrandt et al., 2023). Inadequate IVC conditions responsible for the developmental ‘block’ at the time of embryonic genome activation in novel species has required transfer of embryos in the early cleavage stages in springbok and blesbok (*Antidorcas marsupialis* and *Damaliscus pygargus*, respectively; Chatiza et al., 2013), Eld’s deer (*Rucervus eldii*; Thongphakdee et al., 2017), and reindeer (*Rangifer tarandus*; Lindeberg et al., 2021). In non-mammalian species, yolk-laden oocytes have made cryopreservation significantly more challenging than the smaller mammalian oocytes (Diwan et al., 2020). Despite these setbacks, researchers have not only generated IVF embryos but produced offspring of genetic value to the captive and wild populations.

Spotlight: Brown Brocket Deer (*Subulo gouazoubira*)

Over the past decade, the work of José Duarte on brown brocket deer highlights the challenges in developing IVF programs for novel species. Using a multi-disciplinary approach to neotropical deer genetic preservation involving cytogenetics, molecular genetics, and reproductive biology, Duarte and colleagues have been investigating the full spectrum of ARTs (Rola et al., 2021a). Common brown brocket deer pregnancies were obtained using frozen-thawed sperm, potentially serving as a model for more vulnerable brocket deer species (Duarte et al., 2023). Retrieval of oocytes from super-stimulated ovaries using laparascopic techniques resulted in 65% IVM rates (Rola et al., 2021b). While embryos have not yet been produced in brown brocket deer by conventional IVF methods, iSCNT (using domestic cattle oocytes) resulted in 6% blastocysts (Melo et al., 2022). Notably, progress in wild cervid IVF has been challenging with low blastocyst rates observed in Eld’s deer (5%; Thongphakdee et al., 2017), and reindeer (4%; Peippo et al., 2019). In contrast, dedicated efforts in the Japanese sika deer (*Cervus nippon nippon*), a model for the endangered Vietnamese sika deer (*Cervus nippon pseudaxis*), resulted in 30% blastocyst rates once the embryos were co-cultured with ovine epithelial cells (Locatelli et al., 2012). With so many species in need of assistance, prioritizing resources based on ecosystem or food security relevance will be essential for achieving conservation impact with ARTs.

- Somatic Cell Nuclear Transfer (SCNT)

Following the report by Dominko et al (1999) demonstrating the bovine ooplasm’s capacity as a ‘universal recipient’, interspecies SCNT (iSCNT) emerged, fueling the rise in somatic cell biobanks in zoological and government institutions, as well as the production of iSCNT embryos in more than 50 species (Mastromonaco et al., 2014). Although blastocyst development has been demonstrated in diverse species, including tiger (*Panthera tigris*), red panda (*Ailurus fulgens*), and minke whale (*Balaenoptera acutorostrata*), only a small number of offspring have been born (Mastromonaco et al., 2014). Challenges stemming from low and aberrant reprogramming have impacted the advancement of iSCNT as a practical tool for offspring production. From a species conservation perspective, the recent birth of the black footed ferret clone using donor cells cryopreserved from a wild female in 1988 and oocytes from domestic ferrets provided an opportunity to re-introduce under-represented genetics, thereby having a significant impact on the diversity of the remaining ferret population (Imbler, 2021). This is a key example of the potential for cloning technologies in the One Plan Approach as it was always known that iSCNT would not become a widespread tool for offspring production, but a powerful tool for the ‘resurrection’ of key individuals of high genetic value.

Spotlight: Przewalski’s Horse (*Equus ferus przewalskii*)

Species with small founder gene pools and high inbreeding coefficients typically require reproductive support as with the Przewalski’s horse. Although the first retrievals and interspecies transfers of Przewalski’s horse embryos occurred more than 40 years ago (Allen and Wilsher, 2020), there has been only one reported birth of a healthy foal following timed AI and none from IVPET reviewed by Cabeza and Gambini, 2023). Advancements in domestic horse SCNT (approximately 400 foals born; reviewed by Gambini and Maserati, 2017) fostered the opportunity to investigate iSCNT as a tool to produce Przewalski’s horse foals. Under the guidance of Oliver Ryder with the San Diego Zoo’s Frozen Zoo®, Przewalski’s horse stallion cells cryopreserved in 1980 were used to produce 11 transferrable embryos that resulted in 7 pregnancies and 2 live births (Novak et al., 2024). While it may not be a significant scientific advancement, it is a remarkable change in mind-set as the American Association of Zoos and Aquariums previously did not accept nuclear-cytoplasmic hybrids into the captive gene pool. Further, the project was supported by the not-for-profit Revive & Restore (2024), a sponsor of de-extinction projects such as the woolly mammoth, passenger pigeon, great auk and more (Revive & Restore, 2024), which may be academically interesting pursuits but do not serve the conservation community.

- In Vitro Gametogenesis (IVG)

The potential to ‘convert’ adult somatic cells into induced pluripotent stem cells (iPSCs) using human and mouse transcription factors (‘Yamanaka factors’; Ogorevc et al., 2016) captivated researchers interested in understanding the cellular and developmental biology of wildlife species. Since then, iPSCs have been generated in >20 wildlife species, including snow leopard (*Panthera uncia*), Sumatran orangutan (*Pongo abelii*), little brown bat (*Myotis lucifugus*), Okinawa rail (*Hypotaenidia okinawae*), Japanese ptarmigan (*Lagopus muta japonica*), to name a few (Katayama et al., 2022; Swegen et al., 2023). Advancements in stem cell induction and differentiation brought IVG closer to reality with the birth of mouse pups following in vitro oogenesis that generated the iPSC-derived oocytes (Hayashi and Saitou, 2013). More recently, in vitro spermatogenesis resulted in iPSC-derived spermatids used to produce living offspring in the rat (Matsumura et al., 2023). Similar to SCNT, iPSC-based IVG requires successful reprogramming of adult somatic cells into a stable pluripotent state, a feat that is not easily achieved (Li et al., 2014). Further, differentiation of the iPSCs into primordial germ cell-like cells (PGCLCs) has been effective in the mouse model but difficult to achieve in farm animals (Strange and Alberio, 2023). This comes with additional safety challenges due to the teratogenic potential that arises from using transgene integrating delivery methods and a highly oncogenic set of genes. There is hope that the significant efforts already underway in both the human and animal fields will propel IVG science forward.

Spotlight: White Rhinoceros (*Ceratotherium simum*)

Since the 1990s, cases of ovarian cycle irregularities and reproductive pathologies have been investigated in captive rhinoceros (reviewed by Roth, 2024). However, the plight of the northern white rhinoceros (rhino), a functionally extinct subspecies with only two living adult females, instigated one of the most lucrative partnerships in conservation ART history between BioRescue and Colossal Biosciences (BusinessWire, 2023). What began as technique development to support white rhino breeding programs has resulted in a race to de-extinct the northern white rhino subspecies. Multiple attempts at live and post-mortem oocyte collection followed by IVM-IVF-IVC using domestic horse protocols have recently resulted in blastocysts (Hildebrandt et al., 2023). While there have been 10 white rhino calves produced by AI with fresh and frozen sperm (Roth, 2024), there are no live calves from the transfer of IVF embryos to date. Currently, there are two research teams that have independently induced a pluripotency state in multiple northern white rhino somatic cell lines, some of which had been cryopreserved for 40 years (Korody et al., 2021; Zywitza et al., 2022). Progress towards IVG was reported in 2022 with the generation of primordial germ cell-like cells northern white rhino iPSCs (Hayashi et al., 2022). While these initiatives don’t have immediate application in biodiversity conservation, they are important advancements towards the establishment of somatic cell technologies for genetic management of wildlife species.

## Focus for the Future

Forty years of efforts from researchers around the world have resulted in a proportionately small number of cases that have successfully integrated AI, MOET or IVPET into species conservation plans. To date, ARTs have been more readily applied in external fertilizers, specifically fishes and amphibians, since the in vitro environment does not have the complexity of re-creating the oviductal or uterine milieu ex vivo. In contrast, mammals will not experience the same widespread application and abundance in offspring production anytime soon. There are several key research priorities to continue advancing mammalian ARTs: a) the oocyte (culture optimization and cryopreservation), b) the pregnant uterus (recipient management), and c) the environment (emerging pollutants and climate change). Innovative technologies, ranging from microfluidics to 3-D culture systems (Ferraz and Ferronato, 2023), offer untapped possibilities for overcoming the current limitations in producing embryos with optimal developmental capacity pre- and post-implantation. Similarly, the growing literature on nutrition provides important insights for improving pregnancy maintenance and fetal development (Wyse et al., 2022). Unfortunately, reproductive health and ART success will be challenged by the continuously changing natural environments, particularly ubiquitous particles such as micro- and nano-plastics (Aardema et al., 2024).

As species continue to face threats to their long-term survival, ARTs provide some assurance that the genetics carefully stewarded in wildlife biobanks around the globe can be used to keep keystone species thriving and ecosystems intact. For ARTs to become a realistic addition to the One Plan Approach to species conservation, comprehensive programs must include not only the initial research and protocol development phase, but also establishment of partnerships and generation of banked inventory through systematic sample collection. In a field with such limited resources, it is important to remain focused on species or populations that have the greatest chance for self-sustainability in the long term. Thus, projects directing resources and attention towards extinct or functionally extinct species, while making potentially valuable scientific advancements that could be extrapolated to related species, are a distraction from impact-driven species conservation objectives.
